# Congenital insensitivity to pain and anhidrosis

**DOI:** 10.4103/0019-5413.80047

**Published:** 2011

**Authors:** Ashok H Sasnur, Prakash A Sasnur, Raza Shamikh Muneer Ghaus-ul

**Affiliations:** Department of Orthopaedics, Al Ameen Medical College and Hospital, Athani Road, Bijapur, Karnataka, India

**Keywords:** CIPA, congenital insensitivity to pain, HSAN type IV, malunion, pseudoarthrosis

## Abstract

Congenital insensitivity to pain and anhidrosis (CIPA) is a rare reported entity characterised by disturbance in the pain and temperature perception due to involvement of the autonomic and sensory nervous system. It is an autosomal recessive trait with several defects of the gene *NTRK1* coding for the neurotrophic tyrosine kinase — a nerve growth factor receptor on chromosome 1q21-q22. Traumatic fractures are common and, because of lack of pain, may go unrecognised for prolonged periods, resulting in nonunion or pseudoarthrosis. A Charcot joint may be the end result. Treatment complications are very common in these patients and range from infection to wound breakdown to failure of fixation. We report here a rare case of CIPA in a 9-year-old girl and her younger male sibling with generalised absence of pain, anhidrosis and its orthopaedic implications.

## INTRODUCTION

Congenital insensitivity to pain and anhidrosis (CIPA) is a rare disorder affecting autonomic nervous system, and therefore has been described as Hereditary Sensory and Autonomic Neuropathies (HSAN).^1^ It presents to orthopaedic surgeons with nonunion and pseudoarthrosis following multiple fractures.^2^,^3^ The failure of internal fixation, infection and wound breakdown are common complications. Many of their wounds are largely self-inflicted act of curiosity.

The incidence of this disorder has been estimated to be 1 in 25,000 population.^4^ We report here a rare case of CIPA in a 9-year-old girl and her younger male sibling with generalised absence of pain, anhidrosis and its orthopaedic implications.

## CASE REPORT

**Figure 1:**
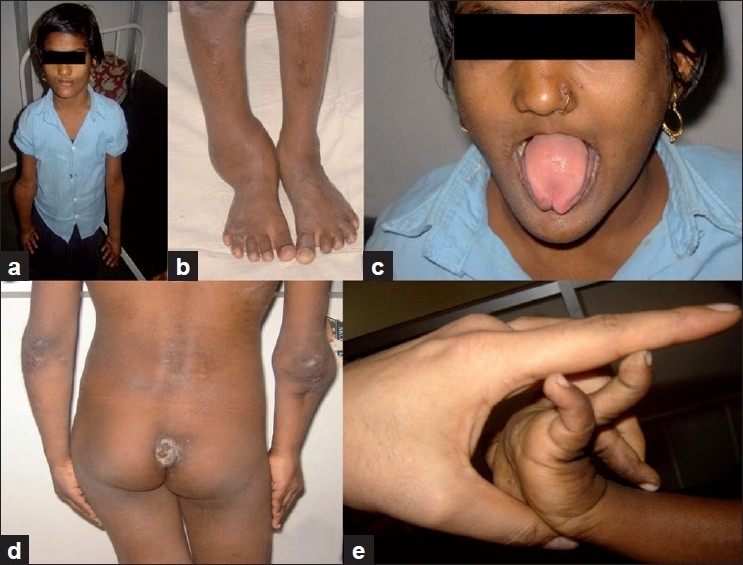
Clinical photograph of the patient shows deformity of the right elbow (a), right ankle (b), bifid tongue (c), multiple scars on the body (d), and ligamentous laxity (e)

A 9-year-old girl presented with a painless and deformed right elbow [Figure 1a] since 5 years and a painless, deformed right ankle [Figure 1b] since 3 months The deformities of joints preceded with history of repeated trauma. Parents brought her with complaints of swelling in her right ankle and limp since 3 months. Over the past years they complain of her being insensate to pain, lack of tears, dry skin, recurring bouts of fever, constipation, biting tongue and ulcers over her back.

**Figure 2:**
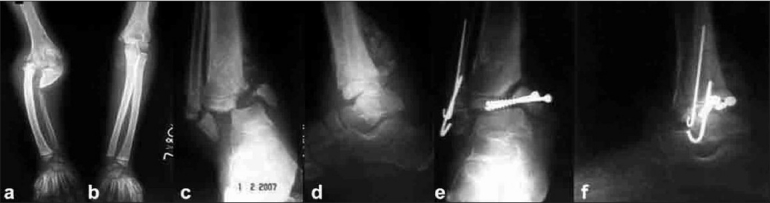
X-ray of both bones forearm with elbow joint (anteroposterior views) (a, b) showing atrophic nonunion of proximal ulna with displaced radial head on right side. X-ray of the right ankle (anteroposterior and lateral views) shows nonunion (c, d), of bimalleolar fracture and internal fixation and for nounion fracture dislocation of right ankle (e,f)

On examination she has generalised absence of pain perception, swollen right proximal forearm with abnormal mobility suggesting nonunion. Her right ankle joint appeared to be medially displaced [Figure 1b]. She had shortening of her right lower limb by 3 cms and had short limb gait. She had a bifid tongue [Figure 1c], absence of most of the teeth, lack of response to painful stimulus, diminished temperature perception, normal touch perception, diminished deep reflexes, fluctuating blood pressure, multiple scars over the body [Figure 1d] and ligamentous laxity [Figure 1e]. Radiographs of her right elbow with forearm revealed atrophic nonunion of proximal ulna with displaced radial head [Figure 2a and b] suggesting old Monteggia fracture dislocation for which she was operated at the age of 6 years at another private hospital. Her right ankle X-rays revealed bimalleolar ankle fracture dislocation [Figure 2c and d].

Her parents are asymptomatic and have history of consanguineous marriage. There is no history of similar complaints from either of the parent’s side. Their son who is seven years old (younger brother of our patient) also has history of tongue bites, lack of tears and insensate to pain but to a very lesser degree and does not present with Orthopaedic manifestations.

Intradermal injection of 0.1 ml of 1:1000 solution of histamine produced a wheal but no pain and no axon flare around it. Nerve conduction velocity was normal, serum creatinine phosphokinase level was elevated and findings of the remaining investigations were within normal limits. The patient was operated at the age of nine years for bimalleolar fracture dislocation. Open reduction and internal fixation with malleolar screws and multiple K-wire was done [Figure 2e and f]. Post-operatively, the limb was immobilised in a below knee slab for 6 weeks. Intra-operative and post-operative recovery was uneventful. However, there were no radiological signs of fracture healing after 6-8 weeks, and she was allowed to ambulate with the help of ankle braces. The fracture did not unite even at one year followup. She had right tibial shortening and managed to walk with limp. At 2 year follow-up she presented with pain and swelling in her right ankle and she was diagnosed as septic arthritis of right ankle joint. She had progressed from pain insensitivity to painful ankle joint, for which she underwent surgical debridement and implant removal. Intra-operatively, implants were buried inside the callus, but fracture resulted in pseudoarthrosis. The nonunion of the proximal right ulna was left alone, as she did not present with any complaints.

## DISCUSSION

Hereditary peripheral neuropathies have been classified based on their clinical characteristics, mode of inheritance, electrophysiological features, metabolic defects, and specific genetic markers.^5^ The most consistent and distinctive feature of an HSAN is loss of pain and perception.

HSAN Type I is most common autosomal dominant inheritance with genetic locus on 9q22.1-q22.3, in *SPTLC1* gene,^6^ characterised by progressive degeneration of dorsal ganglia and motor neurons, leading to distal sensory loss and later in the course of disease, distal muscle wasting and weakness. Symptoms manifests in second to fourth decade of life with slowly progressive numbness, paraesthesias and sensory defects, and plantar ulcers, with less obvious motor deficit.^6^

HSAN Type II is an autosomal recessive trait characterised by loss of pain, temperature, pressure and touch sensation following large and small nerve fibre involvement. This is a disease of infancy and early childhood, with distal numbness in the upper and lower limbs and a glove and stocking sensory loss. The causative gene *HSN2* on chromosome 12q13.33, has been recently reported in five Canadian families.^7^

HSAN Type III or riley day syndrome or familial dysautonomia is one of the many names for this autosomal recessive disorder, which occurs exclusively in Ashkenazi Jewish families. It has an incidence of 27 in 100000 population, but is very rare in other ethnic groups.^8^ The genetic locus has been mapped to chromosome 9^q31–q33^.^8^ These patients present with diminished or absent pain and temperature perception but normal touch perception. They present with anhidrosis, decreased lacrimation, tongue ulcerations, recurrent gastrointestinal (GI) upset, diminished or absent deep tendon reflexes, poorly controlled temperature, a fluctuating blood pressure and an absent axon reflex. Orthopaedic manifestations include gait abnormalities, fractures, charcot joints, osteomyelitis, scoliosis and ligamentous laxity.^8^ There is characteristic absence of fungiform papillae of the tongue and can later develop finger nail dystrophy.^6^

HSAN Type IV is an autosomal recessive disorder associated with several defects of the gene NTRK1 coding for the neurotrophic tyrosine kinase.^6^ This type IV is also called CIPA. First described in 1951, results from a defect in neural crest differentiation and the first-order afferent system responsible for pain and temperature sensation. Clinical suspicion arises when symptoms begin early in infancy, which include profound loss of pain sensitivity leading to injuries, self-mutilation and osteomyelitis.^9^ Loss of oral sensation leads to mutilation of the face and mouth, as seen in our patients. Mild-to-moderate mental retardation, episodic hyperthermia associated with seizures, and anhidrosis are some of the prominent features. Early death from hyperpyrexia occurs in up to 20% of patients, and septicaemia is a frequent occurrence. The histamine test shows no axon flare response, and there is no tear formation and sweating with pilocarpine. Seyon *et al*.^10^ have called CIPA, the “mystery of broken bones” after the patient who was wrongly diagnosed as suffering with osteogenesis imperfecta due to recurring fractures since the age of 4.

HSAN Type V is an autosomal recessive disorder phenotypically similar to HSAN IV presenting with loss of pain and temperature sensation, but other sensations preserved. It is caused by mutation in the nerve growth factor beta gene on chromosome 1. The main difference between HSAN IV and HSAN V was thought to be the pattern of nerve fibre loss, and the greater severity of anhidrosis in HSAN IV and lack of mental retardation in patients with HSAN V.^6^

We were unable to carry out genetic tests, but we believe that diagnosis primarily depends on clinical examinations and specific sensory and autonomic assessments. Our findings on clinical examination are consistent with HSAN Type IV. Although there is no mention of curative treatment in such patients, Bar-On *et al*. describes preventive measures for orthopaedic complications such as the use of special footwear, periods of non-weight-bearing, surgical wide debridement and curative osteotomy for deformity.^8^ Krettek *et al*. encourages to treat initially with non-operative methods and then, if the fracture does not unite on time and there are signs of hypertrophic nonunion, to fix with internal fixation.^11^ Jacob *et al* advices regarding protective footwear, clothing, avoidance of injuries and prompt treatment of wounds and infections is crucial.^6^ Congenital indifference to pain is a problem that is difficult to control and appears impossible to treat, as there are no specific guidelines for fracture treatment. Recurrent injuries can be lessened by preventive measures and vigilant attention by the parents. As described by Drummond *et al*., CIPA patients tend to progress from pain indifference to gradual recovery of pain sensibility.^12^ They strive to be normal as they grow and learn to respond to painful stimuli and hence acquire a pattern of appropriate behavioural responses.^13^

A rare case of HSAN Type IV, CIPA showing the usual features of biting lips, tongue and self-mutiliation, repeated fractures, nonunions with anhidrosis is presented here. Patient initially presented with indifference to pain and later progressing to gradual recovery of normal pain sensibility. Both fractures resulted in nonunion despite adequate operative interventions.
